# Tobacco and Alcohol Use and the Impact of School Based Antitobacco Education for Knowledge Enhancement among Adolescent Students of Rural Kerala, India

**DOI:** 10.1155/2016/9570517

**Published:** 2016-08-17

**Authors:** Radhakrishnan Jayakrishnan, Seema Geetha, Jagathnath Krishna Kumara Pillai Mohanan Nair, Gigi Thomas, Paul Sebastian

**Affiliations:** ^1^Division of Community Oncology, Regional Cancer Centre, Thiruvananthapuram, Kerala 695011, India; ^2^Department of Periodontics, Sri Sankara Dental College, Akathumuri, Vennicode, Thiruvananthapuram, Kerala 695318, India; ^3^Division of Cancer Epidemiology and Biostatistics, Regional Cancer Centre, Thiruvananthapuram, Kerala 695011, India; ^4^Regional Cancer Centre, Thiruvananthapuram, Kerala 695011, India

## Abstract

*Objectives*. Limited information is available on adolescent tobacco and alcohol use in rural Kerala, the southernmost state in India. The study was conducted to estimate the prevalence of tobacco and alcohol use among adolescent school students and further to understand the extent of knowledge pertaining to tobacco before and after conducting awareness programmes in schools.* Methods*. A cross-sectional study was conducted in 10 government schools of rural Thiruvananthapuram district of Kerala state based on a multistaged sampling design. Using a pretested semistructured questionnaire, prevalence and patterns of tobacco use by students and their households, as well as students' knowledge on tobacco hazards before and after delivering antitobacco messages, were collected.* Results. *The overall prevalence of self-reported ever users of tobacco in the current academic year was 7.4% (95% CI 5.86–8.94), while that of ever alcohol users was 5.6% (95% CI 4.25–6.95). Knowledge assessment scores revealed a significant increase in the mean knowledge scores after posttraining evaluation (mean score = 10.34) when compared to pretraining evaluation (mean score = 9.26) (*p* < 0.0001).* Conclusion*. Apart from antitobacco awareness programmes, strict monitoring of trade of tobacco and alcohol products near educational institutions has to be conducted consistently to curb the problem.

## 1. Introduction

One of the most widely discussed public health problems in the world is the health impact of tobacco use, which kills more than 6 million people globally every year. The mortality due to tobacco is more than that associated with tuberculosis, HIV/AIDS, and malaria combined [1]. Tobacco related diseases, particularly cancers, have become nightmarish to man in recent decades and continue to lead the table among the top ten male cancers in India [2]. The dreadful issues range from suffering and disfigurement due to oral cancer at one end [3] to the dreaded lung cancer with its high mortality rates at the other end [2, 4]. Additionally, approximately 10 different types of cancers with varying prognosis have been found to have a direct or indirect link to tobacco habits [5]. While these remain the alarming facts about tobacco related cancers, the list of tobacco related diseases other than cancers number more than a dozen, virtually affecting every organ of the body [6].

Among the 1 billion smokers in the world, nearly 80% live in low and middle income countries. In India, it is estimated that more than one-third of the population aged 15 years and above are current tobacco users. Over a million tobacco related deaths are reported annually in India [7]. The figures are alarming for a country like India which has the world's largest youth population [8]. Currently the tobacco companies aggressively target young population of the developing countries to market tobacco products.

India's tobacco control measures are complex because of the large population, easy availability of inexpensive tobacco products, and disproportionate implementation of tobacco control laws particularly in the rural areas [9].

Today, the single greatest opportunity for preventing noncommunicable disease in the world points to the prevention of tobacco use in young people [10]. Improvement of tobacco control measures, particularly targeting the youth, is important because of its long term impact in adulthood. It is understood that tobacco addiction among the majority of adults has been initiated during their adolescence period [11]. It is estimated that nearly three quarters of premature deaths among adults are due to behavioural patterns that emerge during adolescence, including smoking, violence, and sexual behaviour [12]. The Global Youth Tobacco Survey (GYTS), a cross-sectional survey conducted among school students, aged 13–15 years, highlighted that the overall prevalence of current tobacco users in India was 14.6% in the year 2009. The GYTS also pointed out that second hand smoke exposure was found among one in five students at homes, where others smoked [13]. Similar to tobacco, alcohol consumption during adolescent period could have profound implications in health, risking brain development, and violence and was found to be a significant predictor for alcohol dependence in adulthood [14, 15].

The state of Kerala, located in the south west corner of India, also suffers from the tobacco menace in spite of its high literacy and better health care systems. The Global Adult Tobacco Survey reports that the prevalence of any form of tobacco use in Kerala among the adult male population is 35.5% [7]. Though smoking is the predominant habit among adult males, smokeless tobacco forms are popular among school students. Smokeless tobacco forms containing areca nut (a seed of the* Areca catechu* palm) are the major causative factor for oral submucous fibrosis, an oral potentially malignant disorder [16]. Gutkha and pan masala products are widely used in India. Gutkha is an addictive form of smokeless tobacco that contains crushed areca nut, tobacco, catechu, paraffin wax, and slaked lime along with flavouring agents, while pan masala is a generic term used for marketing and promotion of products with the ingredients of gutkha excluding tobacco [17, 18]. Betel quid chewing (betel quid is a combination of betel leaf, areca nut, and slaked lime and may contain tobacco) is popular in the rural areas of Kerala. The comprehensive tobacco control law in India (Cigarettes and Other Tobacco Products Act 2003) has explicitly described the implications of selling tobacco products to minors in section  6 of the act. Section  6 (a) of the act clearly describes the prohibition of sales to minors and section  6 (b) focuses on the prohibition of sale of tobacco products within a radius of 100 yards of any educational institution [9, 19]. In addition to this, the Government of Kerala, based on the Food Safety and Standards Act 2006, has imposed a complete ban on manufacture, storage, and sale of gutkha and pan masala containing tobacco and/or nicotine in the state [20]. Recently, Kerala has become the first state in India to be declared tobacco advertisement free, an achievement which was made possible because of a favourable political commitment, strong policy framework, and an active engagement of various agencies in the state [21].

The information on tobacco and alcohol use among school students from rural Kerala is limited. A study conducted among high school students in the northern district of Kannur in Kerala reported 8.5% prevalence of smokeless tobacco use, whereas the prevalence of any type of tobacco use reported in the rural areas of Central Kerala was 7.5% [22, 23].

Very few studies were published from Kerala regarding alcohol consumption among adolescents. A recent study conducted in Ernakulam in Kerala among school students reported 15% prevalence in the 12–19-year-age group [24].

Information on the prevalence of tobacco use after strengthening tobacco legislation has not been reported in Kerala. Similarly there is very limited information on the alcohol use prevalence among adolescents. In addition to this, there is inadequate information on the influence of family or household members on tobacco and alcohol use among students and the impact of antitobacco education on enhancing knowledge on tobacco hazards. It was reported that antitobacco awareness programmes play a major role in enhancing awareness and modifying the attitude of adolescent school students towards tobacco use [25]. It is against this backdrop that the current study was conducted to estimate the prevalence of tobacco and alcohol use among high school and higher secondary school students in rural Kerala and further to understand the extent of knowledge pertaining to tobacco before and after conducting sensitisation programmes in schools.

## 2. Materials and Methods

### 2.1. Study Area

The study was conducted in the Thiruvananthapuram district located in the southern part of Kerala. The city of Thiruvananthapuram is the district headquarters and also the capital of the state of Kerala. For administrative purpose, each revenue district in the state was divided into educational districts and further into educational subdistricts. The study was conducted in rural Thiruvananthapuram district. In Thiruvananthapuram district there are 3 educational districts and 12 educational subdistricts. Each educational district has approximately 40 high school/higher secondary schools listed in the government school category.

### 2.2. Study Design and Methods

A multistage sampling design was adopted for selecting the study units. In the initial phase, among the 40 government schools in one educational district, one educational subdistrict was randomly chosen for the study. This educational subdistrict was located in a rural area. There were 17 schools in this educational subdistrict. 10 schools were selected randomly using computer generated random numbers. The sampling unit in the study was the class division. Most of the class divisions had strength of 35 to 50 students. Information was collected from each student before and after delivering antitobacco awareness sessions.

The Institutional Review Board of the Regional Cancer Centre, Thiruvananthapuram, had accepted the study protocol prior to the commencement of the study. The rationale for conducting the study was explained to the study participants and their participation in the study was purely voluntary. Verbal informed consent was obtained from each participant.

#### 2.2.1. Tobacco and Alcohol Prevalence

The study attempted to understand the prevalence of “ever user” in the current academic year, that is, whether the person had used tobacco or alcohol at least once during the academic time period when the study was conducted. Similarly, a “current tobacco user” was defined as a person who had the habit of using tobacco, 3 days or more a week. This definition was also applied for estimating alcohol prevalence.

### 2.3. Inclusion/Exclusion Criteria

Eligible subjects included male students of classes IX and X belonging to the high school (HS) group while XI and XII standards were included in the higher secondary school (HSS) category. Male students were enrolled for the study due to the fact that tobacco use was reported only among males in Kerala state. Government schools were included in the study due to the fact that majority of the students studying in these schools are from the lower socioeconomic strata and also because it was expected that tobacco and alcohol consumption could be relatively high among them.

### 2.4. Tools for the Study

#### 2.4.1. Pretraining and Posttraining Questionnaire

The impact of antitobacco awareness programme on enhancing the knowledge of subjects was evaluated based on the information collected from the pretraining and posttraining questionnaire. A pretested semistructured questionnaire was used for the study. The questionnaire was pretested at two selected government schools after which the necessary changes were made to ensure that the questionnaire was easy to understand and could be answered within a short period of time. Later, this questionnaire was used for pre- and posttraining evaluation in the selected schools. The questionnaire had two sections. In the first section of the questionnaire, apart from age and the class in which the student is studying, questions to elicit the tobacco and alcohol usage habits of students and their household members and also the sale of tobacco and alcohol near educational institutions were included. The second section of the questionnaire was used for pretraining and posttraining evaluation. This section consisted of questions related to the knowledge on tobacco hazards, tobacco control law in India, and also the common ingredients in tobacco. Knowledge scores were calculated for each subject before the intervention (pretraining evaluation) and after intervention (posttraining evaluation). The maximum knowledge score a subject could achieve was 16. The evaluation test consisted of 4 open ended and 12 true-false type questions. The questionnaire was explained by the research team specifically clarifying each question, thereby ensuring that maximum information could be elicited from the participants. The students were also assured that anonymity regarding the information provided would be maintained and that the information would not be shared with the team members. To avoid the fear of getting noticed by the school staff, while answering the questionnaire, staff members were requested to leave the venue till the students answered and returned the questionnaire to the study team.

A unique number was provided to each set of questionnaires and the respondents were told not to reveal their names. The purpose was to increase participation in the study. Once the questions were discussed by the study team member, students were asked to complete the questionnaire within fifteen minutes. The pretraining forms were collected by the team member and an awareness seminar in each school followed. After completing the awareness seminar, posttraining evaluation forms were filled and returned.

#### 2.4.2. Antitobacco Awareness Programmes

Resource persons conducted awareness programmes on tobacco hazards in schools. For each school, one awareness programme was conducted using audio visual slides. Awareness programmes focused on the magnitude of the problem in India, diseases due to tobacco, the scientific knowledge on ingredients in tobacco, tobacco addiction, and the tobacco control laws in India. The awareness programmes were conducted in two sessions in a day separately for high school and higher secondary school students. A short film on tobacco hazards developed by the Regional Cancer Centre, Thiruvananthapuram, was also screened at the end of each session.

### 2.5. Data Analysis

Statistical analysis was done using the SPSS version 16 software. For categorical variables, Chi-square statistics were used. Fisher's exact test statistics were used if the expected value of a cell was less than five. For continuous variables, to test mean differences between more than two groups, Analysis of Variance Test (ANOVA) was used and, for those significant variables, Bonferroni multiple comparison test was used to find the significantly differing groups. To compare the mean knowledge score of participants before and after conducting awareness programmes, paired sample *t*-test was used.

## 3. Results

### 3.1. General Characteristics of the Study Subjects

A total of 1200 self-reporting questionnaires were distributed to the students for the study. The participation in the study was 89% with 1114 students responding by returning the filled-up questionnaire. The mean age of the students was 16 years (SD 3.3). Within the study subjects, 539 students were from HS category while the remaining 575 students belonged to the higher secondary school (HSS) category.

### 3.2. Prevalence of Tobacco and Alcohol Use

The overall prevalence of self-reported ever users of tobacco in the current academic year was 7.4% (95% CI 5.86–8.94) while current users constituted 4.3% of the study subjects (95% CI 3.11–5.49). The prevalence of tobacco use increased with increase in age (*p* = 0.0001) (Table 1).

Cigarette smoking was the predominant habit among ever users (2.8%) followed by gutkha (2.2%), beedi smoking (1.3%) (beedi is locally made by casing coarse tobacco), and betel quid chewing (1.2%). In the present study, the proportion of ever users of alcohol was 5.6% (95% CI 4.25–6.95) while current alcohol use was reported by 0.8% of the study subjects. A significant difference was observed in the prevalence of tobacco use among HS and HSS groups. While the prevalence of ever users of tobacco in the HS group was 5.5%, the corresponding figures for HSS group were 9.2% (*p* = 0.012). Similarly for alcohol, ever users of alcohol in the current academic year constituted 5.6% (95% CI 4.25–6.95) of the subjects. The prevalence was high among the HSS group (4.5%) when compared to the HS group (1%).

### 3.3. Influence of Household Member's Tobacco and Alcohol Habits on Adolescent Users

The study also looked into the association between tobacco use among students and household members with tobacco habit. Among ever users of tobacco in the current academic year, it was reported that 63% of their household members used tobacco in some form or the other (*p* = 0.0001) (Table 2). Similarly, alcohol use among household members was also found to be significantly associated with study subjects falling under the category of ever users of liquor (*p* = 0.003).

### 3.4. Perception on Tobacco and Alcohol Sales near Educational Institutions

When asked about the sale of tobacco within a radius of 100 yards of educational institutions, nearly 55% of the subjects reported tobacco sales near educational institutions. Significantly higher percentage of students (64%) in class 12 category reported tobacco sales near schools, while the lowest reported was those in the class 9 category (40%) (*p* = 0.0001). However, no significant difference was found when ever users and never users of tobacco were compared (*p* = 0.291). Regarding the sale of alcohol near schools, 89% were not aware of alcohol sale near educational institutions, though majority of those who reported alcohol sales were from the higher secondary school category (*p* = 0.014) (Table 3). While ever and never users of alcohol were compared, no significant difference was observed (*p* = 0.068) as far as alcohol sales near schools were concerned.

### 3.5. Response of Subjects to Banned Tobacco Advertisement Boards

When enquired about having noticed tobacco advertisement boards in their vicinity, 70% of students in the HS group and 77% in the HSS group reported having seen tobacco advertisement board during the last one month of conducting the study. Overall nearly three quarters of the study subjects had seen tobacco advertisement boards during this period.

### 3.6. Knowledge on Tobacco Hazards and Antitobacco Law

The knowledge on tobacco hazards and the law restricting smoking in public places was assessed among high school and higher secondary school students, before awareness programmes were conducted (Table 4). When enquired about the law restricting smoking in public places, the HSS students had higher awareness (92%) on the law when compared to HS students (82.3%) (*p* = 0.001). Similarly, a significant difference was observed between HSS students and HS students regarding awareness on the harmful effects of pan masala, where the former group reported more awareness on pan masala hazards (*p* = 0.031). Regarding awareness on the harmful effects of passive smoking (awareness among HS = 75.9% versus HSS = 79.8%) and the addictive nature of pan masala (HS = 92% versus HSS = 93.5%), no significant difference was observed between the two groups. We also looked into the knowledge of students regarding the basic ingredients of tobacco with reference to nicotine and tar. 79% of HS students and 66% of students in the HSS group were unaware of nicotine and tar in tobacco.

### 3.7. Comparison of Pretraining and Posttraining Analysis

**Figure 1:**
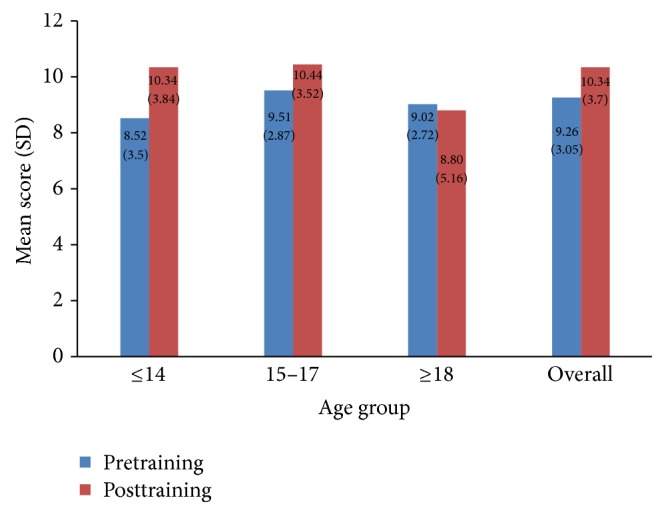
Comparison of pretraining and posttraining knowledge scores.

Overall, the mean knowledge score of the study subjects after pretraining evaluation was reported to be 9.26 while the posttraining evaluation noted that the mean knowledge had marginally increased to 10.34 (*p* < 0.001) (Figure 1). When the mean knowledge scores were compared between ever users and never users of tobacco, no significant difference was observed between the groups in the pretraining session (never users = 9.30 versus ever users = 8.72, *p* = 0.096). However, posttraining evaluation scores showed a significant difference (never users = 10.49 versus ever users = 8.47, *p* < 0.001). Comparisons within the group had noted that, among never users, the difference in mean knowledge scores before and after intervention was significant (9.30 versus 10.49; *p* value < 0.0001), while no such difference was observed among subjects who were ever users of tobacco (8.72 versus 8.47; *p* value = 0.584). When class-wise comparison of knowledge scores was analysed, a significant difference in mean scores between HS and HSS students (8.85 versus 9.65, *p* = 0.001) was found during the pretraining evaluation while no such difference was observed in posttraining scores (10.35 versus 10.34, *p* = 0.95). The age-wise comparison of knowledge scores during pretraining evaluation showed significant difference between the age groups. However the Bonferroni multiple comparison test revealed that the significant difference was between ≤14-year-age and 15–17-year-age groups (*p* < 0.001). Similarly knowledge score during posttraining also showed a statistical significance among the groups (≤14 years = 10.34, 15–17 years = 10.44, and ≥18 years = 8.80; *p* = 0.010). On the other hand, the Bonferroni multiple comparison tests had shown that the significant difference existed between the age groups of ≤14 and ≥18 years (*p* = 0.021) and 15–17 and ≥18 years (*p* = 0.007).

## 4. Discussion

The present study was conducted in the year 2014-2015 to understand the prevalence of tobacco use among adolescent school students in rural Thiruvananthapuram district of Kerala state. The study also evaluated the effectiveness of antitobacco education programmes enunciated in schools to enhance the knowledge on tobacco hazards among students based on the pre- and posttraining questionnaires. In the present study, the prevalence of ever users of tobacco in the “current academic year” was 7.4% of which 4.3% were “current tobacco users.” The proportion of ever users of alcohol was 5.6% of which 0.8% was current users. With reference to knowledge enhancement on tobacco control, the mean knowledge score before (pretraining) and after (posttraining) antitobacco education programmes conducted in schools reported a significant difference (pretraining = 9.26, posttraining = 10.34).

Tobacco use initiated during adolescence could emerge as a major risk factor for noncommunicable disease in adulthood [26]. Hence understanding the magnitude of tobacco use among adolescents assumes significance for timely intervention to curb the problem. The Global Youth Tobacco Survey reported that the likelihood of students taking up tobacco in India would be 15.5% annually [13]. In a study conducted in the southern state of Tamil Nadu in India, the prevalence of any form of tobacco use was 7.1% [27]. The current study results are in line with the youth tobacco survey conducted in Tamil Nadu. When compared to the GYTS India report, the prevalence of current tobacco users in this study was low (4.3%). However, careful interpretation of the study results showed that ever users of tobacco use in the current academic year were 7.4% which needs to be related to the current tobacco control measures adopted in Kerala, where coordination of various departments to augment tobacco control measures had geared up recently. The current study was conducted in rural Kerala from where limited information was reported on tobacco use. Though there was a marginal difference in the cigarette (2.8%) and gutkha habits (2.2%) among students, it was important to note that in spite of the ban on pan masala containing gutkha, its use was equally prevalent among students as cigarettes. This study assumes significance in a state like Kerala where antitobacco polices have been strengthened in recent years particularly from the year 2012 when pan masala containing gutkha was banned in Kerala. One of the most important measures to curb tobacco use which was found to be effective in developed and low income countries was to increase the tax on tobacco products. This could promote tobacco cessation or reduce the quantity of consumption among current users and dissuade youngsters from picking the habit [28, 29]. The Government of Kerala had increased the value added tax (VAT) of cigarettes in the years 2012-2013, 2013-2014, and 2014-2015 to 15%, 20%, and 22%, respectively [30]. The VAT imposed on cigarettes was further raised to 30% in the year 2015-2016. The revenue collection through sales tax/VAT increased from approximately 61 million US$ in the year 2012-2013 to 90 million US$ in the financial year 2013-2014. Beedi was taxed at 14.5% for the first time in the history of Kerala state in the year 2015 [31]. It was against this backdrop that the study was conducted to find out whether a change in tobacco prevalence was observed among adolescent school children in rural Kerala.

When compared to the results reported from previous studies conducted in Kerala particularly in the central (7.5%) and northern districts (8.5%), it has been noted that there was no steady decline in tobacco consumption among “ever users” in the current academic year (7.4%). However, the self-reported current users (3 days or more of tobacco consumption) constituted 4.3% of the study subjects (95% CI 3.11–5.49). The compliance with provisions regarding the sale of tobacco products is moderate in the present study. The study results point to the inadequate enforcement of ban on sale of tobacco within 100 yards of educational institutions. Overall, 60% of students in the HSS group and 52% in the HS group reported illegal sale of tobacco near educational institutions. Tobacco sale within 100-yard radius of educational institutions is prohibited under Cigarettes and Other Tobacco Products Act, 2003 (COTPA). The study findings reflect on the illegal sale of tobacco products near educational institutions which was known probably more among students rather than the public and hence identifying the source of sale of tobacco products should emerge as a challenge for the law enforcers. Similar results were observed in a study conducted in the National Capital Region which also reported violation of tobacco control laws in many educational institutions near the Indian Capital State of New Delhi [32].

In the present study, influence of tobacco habits in households was found to be significantly higher among ever users of tobacco. Efforts targeting adolescent tobacco use should also take into consideration other factors like the tobacco use in households. Nonsmoking parents could be an influential factor for adolescents not to take up the habit; however it would be the reverse if one of them is a tobacco user [33]. Studies had reported that adolescents might perceive tobacco use as an acceptable social behaviour if any of their family members had the habit [10]. Similar observation was noted for alcohol consumption as well. This points to the need to sensitise the family members and households on their health risk behaviour and its influence among adolescents. A study conducted among students aged 12–19 years in Kerala reported a grave situation where school students initiated alcohol consumption because of the extended support of the family members to drink in the pretext of family celebrations [24]. This study also reported high prevalence of adolescent alcohol consumption (15%) when compared to the present study where only 5.6% subjects were ever users of alcohol. A possible explanation for this was probably the greater sample size (more than 7000 from 73 schools) and a wider age group (12–19 years) when compared to the present study. Moreover the former study was conducted in a district which is the commercial hub of Kerala where rapid urbanisation is taking place. Due to its impact, the emergence of drinking as a perceived social custom might have influenced the study population and eventually the results. Taking into consideration the rise in tobacco and alcohol use among school students, multiple approaches at different levels, namely, the school and in the community, could be adopted to enhance tobacco and alcohol control measures.

The impact of antitobacco awareness programmes before and after conducting awareness programmes was assessed in the study. Overall, the study had shown that the mean knowledge scores of study subjects were significantly higher after conducting awareness programme, though the difference in knowledge scores is small. The knowledge scores were less for HS students in the pretraining session when compared to the HSS group; however no such difference existed after posttraining evaluation. The findings show the potential of antitobacco awareness programmes in increasing awareness among school students on tobacco hazards. Another interesting observation was that, among never users of tobacco, a significant difference was observed in the knowledge scores after conducting the awareness programme, while surprisingly no such difference was noted among the ever users of tobacco group. The possible reason for the striking contrast in the results could be that the ever user group were either less attentive or in a state of denial regarding the facts mentioned in the awareness programme. This indicates that more concerted and coherent efforts are required to make a significant impact in the knowledge and attitude on tobacco hazards in this group.

## 5. Limitations 

The study was conducted in one educational subdistrict of the state of Kerala and therefore cannot be generalised as reflecting on the conditions that exist in the entire state. The results were based on self-reported questionnaires and hence both underreporting and overreporting might be possible. Only government schools were selected for the study which may not be a true representation of the total tobacco and alcohol prevalence among adolescents. Both pretraining and posttraining evaluation were conducted on the same day when the awareness programme was conducted. Hence the long term influence of awareness programmes was not extrapolated in the study. The study was restricted to male students due to the reason that tobacco and alcohol use were a predominant problem among adolescent males in Kerala, while females were excluded from the study which needs to be studied as a separate entity altogether.

## 6. Conclusion

Adolescent tobacco and alcohol use in rural Kerala is a matter of concern. Adolescent health education, targeting tobacco and alcohol in particular, should be implemented in schools and has to be continued systematically. Sensitisation programmes for adolescent students and members of Parent Teacher Association have to be given in a sustainable manner. Availability of tobacco and alcohol products particularly near educational institutions needs to be checked regularly by the law enforcers and stringent measures adopted to make certain that such crimes are not repeated. In short, it is recommended to adopt multipronged strategies that encompass all these measures to prevent availability of tobacco and alcohol products near educational institutions and thereby restricting its accessibility to users and initiation among nonusers.

## Figures and Tables

**Table 1:** Distribution of never and ever users of tobacco in the current academic year based on age group and education.

Factors	Never users (*n* = 1031)	Ever users in the current academic year (*n* = 83)	Total	*p* value
Age group				
≤14 years	254 (24.6)	6 (7.2)	260 (23.3)	0.0001^*∗*^
15–17 years	738 (71.5)	66 (79.5)	804 (72.2)	
≥18 years	39 (3.7)	11 (13.3)	50 (4.5)	

Tobacco use among students based on class division				
Class IX	191 (18.5)	9 (10.8)	200 (18)	
Class X	320 (31)	19 (22.9)	339 (30.4)	0.019^*∗*^
Class XI	218 (21.1)	21 (25.3)	239 (21.5)	
Class XII	302 (29.3)	34 (41)	336 (30.2)	

^*∗*^Statistically significant at 5% level using Chi-square test. Figures in parenthesis are column percentages.

**Table 2:** Association between tobacco use and household member using tobacco or alcohol.

Household member using tobacco and alcohol	Never users (*n* = 1031)	Ever users (*n* = 83)	Total (*N* = 1114)	*p* value
Tobacco use among household				
Yes	381 (37)	52 (62.7)	433 (39)	0.0001^*∗*^
No	650 (63)	31 (37.3)	681 (61)	

Alcohol use among household				
Yes	342 (33.2)	46 (55.4)	388 (35)	0.0001^*∗*^
No	689 (66.8)	37 (44.6)	726 (65)	

^*∗*^Statistically significant at 5% level using Chi-square test. Figures in parenthesis are column percentages.

**(a):** 

Category	Tobacco sales not seen	Tobacco sales noticed	Total	*p* value
Class IX	120 (60)	80 (40)	200 (100)	
Class X	146 (43.1)	193 (56.9)	339 (100)	
Class XI	115 (48.1)	124 (58.9)	239 (100)	0.0001^*∗*^
Class XII	122 (36.3)	214 (63.7)	336 (100)	

Total	503 (45.2)	611 (54.8)	1114 (100)	

**(b):** 

Category	Alcohol sales not seen	Alcohol sales noticed	Total	*p* value

Class IX	187 (93.5)	13 (6.5)	200 (100)	
Class X	312 (92)	27 (8)	339 (100)	
Class XI	205 (85.8)	34 (14.2)	239 (100)	0.014^*∗*^
Class XII	287 (85.4)	49 (14.6)	336 (100)	

Total	991 (89)	123 (11)	1114 (100)	

^*∗*^Statistically significant at 5% level using Chi-square test. Figures in parenthesis are row percentages.

**Table 4:** Student's knowledge and attitude on tobacco hazards.

Category	HS students (*n* = 527)		HSS students (*n* = 571)		*p* value
	No	Yes	No	Yes	
Passive smoking harmful	114 (24.1)	359 (75.9)	101 (20.2)	398 (79.8)	0.085
Smoking in public place punishable	91 (17.7)	424 (82.3)	44 (8)	508 (92)	0.001^*∗*^
Pan masala harmful	81 (15.8)	432 (84.2)	65 (11.7)	491 (88.3)	0.031^*∗*^
Pan masala addictive	39 (9.8)	461 (92.2)	36 (6.5)	515 (93.5)	0.249

^*∗*^Statistically significant at 5% level using Chi-square test. Figures in parenthesis are column percentages.
